# Different levels of brain-derived neurotrophic factor and cortisol in healthy heavy smokers

**DOI:** 10.1590/1414-431X20176424

**Published:** 2017-10-19

**Authors:** C.D.C. Neves, A.C.R. Lacerda, L.P. Lima, V.K.S. Lage, C.H. Balthazar, H.R. Leite, V.A. Mendonça

**Affiliations:** 1 Programa Multicêntrico de Pós-Graduação em Ciências Fisiológicas, Sociedade Brasileira de Fisiologia, Universidade Federal dos Vales do Jequitinhonha e Mucuri, Diamantina, MG, Brasil; 2 Laboratório de Inflamação e Metabolismo, Universidade Federal dos Vales do Jequitinhonha e Mucuri, Diamantina, MG, Brasil

**Keywords:** BDNF, Nicotine consumption, HPA axis, Addiction, Reward system

## Abstract

Studies suggest that brain-derived neurotrophic factor (BDNF) and the hypothalamic-pituitary-adrenal (HPA) axis modulate dopaminergic activity in response to nicotine and that the concentrations of BDNF and cortisol seem to be dependent on the amount and duration of smoking. Therefore, we investigated BDNF and cortisol levels in smokers ranked by daily cigarette consumption. Twenty-seven adult males (13 non-smokers and 14 smokers) participated in the study. The smokers were divided in two groups: light (n=7) and heavy smokers (n=7). Anthropometric parameters and age were paired between the groups, and plasma BDNF and salivary cortisol levels were measured. Saliva samples were collected on awakening, 30 min after awakening, at 10:00 and 12:00 am, 5:00 and 10:00 pm. Additionally, cotinine serum levels were measured in smokers. Heavy smokers had higher mean values of BDNF compared to the control group (P=0.01), whereas no difference was observed in light smokers. Moreover, heavy smokers presented lower cortisol levels in the last collection (10:00 pm) than the control group (P=0.02) and presented statically higher values of cotinine than the light smokers (P=0.002). In conclusion, changes in BDNF and cortisol levels (10:00 pm) appear to be dependent on heavy cigarette smoking and can be involved in activation and in the relationship between the mesolimbic system and the HPA axis.

## Introduction

Cigarette smoke is a mixture of gases and particles, and nicotine is the main addictive substance in tobacco. After each inhalation, nicotine rapidly reaches the brain, where it acts primarily on dopaminergic neurons in the mesolimbic system (reward system), leading to dopamine release, which is responsible for euphoria and pleasure feelings and constitutes the key component of addiction (1).

Brain-derived neurotrophic factor (BDNF) is a key molecule related to the modulation of this system. BDNF is one of the most abundant neurotrophins in the brain and it is involved in neuronal growth, differentiation and survival. Moreover, BDNF has been identified as a regulator of synaptic plasticity (2), which is responsible for enhancing dopamine release and responsiveness in the mesolimbic system (3). Thus, evidence has suggested that BDNF modulates the rewarding effects of addictive drugs, among them nicotine (4). Additionally, the BDNF upregulation could be related to the amount and duration of smoking (5).

In addition to BDNF, studies have demonstrated that the hypothalamic-pituitary-adrenal (HPA) axis also seems to mediate dopaminergic activity (6,7), influencing the nicotine consumption of smokers. Similarly, it has been demonstrated that changes in dopaminergic activity also have an effect on glucocorticoid levels (7). In addition, the concentrations of cortisol in smokers seem to be dependent on the number of cigarettes smoked per day (8), suggesting a dose-dependent effect of nicotine or a threshold response to nicotine in activating the HPA axis (9).

Therefore, because both BDNF and the HPA axis appear to modulate dopaminergic activity in response to nicotine exposure, this study simultaneously evaluated central (BDNF) and peripheral (cortisol) components related to nicotine consumption by smokers. Furthermore, since the levels of both BDNF and cortisol in smokers seems to be dependent on the amount and duration of smoking, we proposed to investigate BDNF and cortisol levels in healthy smokers, stratified by daily cigarette consumption; previous studies have not yet been carried out. Therefore, this pilot study aimed to evaluate the BDNF and cortisol levels in healthy adult smokers, ranked by daily cigarette consumption and compared to healthy non-smokers.

## Material and Methods

### Subjects

Thirty-five healthy adult men (17 non-smokers and 18 smokers), aged 18–45 years, were recruited by personal invitation during visits to homes and health centers in the local community. To be included in the study, the subjects had to meet the following criteria: normal lung function; self-report of no current acute or chronic diseases; be eutrophic according to the body mass index (BMI between 18.5–24.9 kg/m^2^); not currently using anti-inflammatory medications; self-reported absence of cough, infection, fever and flu in the month prior to the assessments; and no use of nicotine replacement therapy in the last three months. Control subjects could not have been passive smokers and cigarettes used by smokers must have been manufactured with a filter. The exclusion criteria were the lack of normal circadian rhythm: salivary cortisol at 10:00 pm <6.0 nmol/L and an 8:00 am/10:00 pm ratio of salivary cortisol >2. This study was performed according to Resolution No. 466/12 of the National Health Council. The Research Ethics Committee of the Universidade Federal dos Vales do Jequitinhonha e Mucuri, Brazil, approved this study (protocol No. 003/12). All participants gave written informed consent.

### Procedures

Subjects went to the laboratory for clinical assessment, which consisted of body composition and lung function measurements and the recording of the smoking history of smokers. The body composition was evaluated by BMI, calculated as the weight divided by the square of the height, and by body fat percentage estimated by skinfold thickness using a plicometer.

Lung function was measured using a digital spirometer (PonyFX®, COSMED, Italy). The forced expiratory volume in 1 s (FEV_1_), forced vital capacity (FVC) and FEV_1_/FVC were calculated in accordance with the American Thoracic Society/European Respiratory Society (10).

Smoking history was determined through self-report of the number of cigarettes smoked per day and the number of pack-years, calculated as the number of smoked cigarettes per day/20 and multiplied by the numbers of years of smoking (11). To rank the subjects by daily cigarette consumption, the smokers were divided into two groups. The first group was composed of light smokers, defined as those that smoked up to 10 cigarettes/day, and the second group was composed of heavy smokers, defined as those that smoked more than 10 cigarettes/day (11). One week after the first assessment, the subjects performed the second and third evaluations of the study, described below.

### Cortisol assessment

One week after the clinical assessment, the subjects underwent cortisol evaluation. Because the cortisol level increases 30 min after awakening and declines throughout the day, multiple measurements of cortisol levels during the day provide a more valid information regarding the daily cortisol release and reflect cortisol circadian rhythm. Saliva samples were, therefore, collected upon awakening and after 30 min, at 10:00 and 12:00 am, and 5:00 and 10:00 pm. For this purpose, the participants were instructed to collect saliva samples using a Salivette¯ (Sarstedt, Germany). Volunteers were instructed not to smoke before the first collection (immediately upon awakening) and to avoid smoking for at least 30 min before each collection throughout the day. In addition, they were instructed to avoid alcoholic beverages, physical activity and eating 30 min before sample collection and brushing their teeth 2 h before sample collection. All collections were performed in the subject's house. After each collection, the subjects stored the Salivettes at –20°C. On the following day, the researchers transported the samples under refrigeration to the laboratory, and after centrifugation (1500 *g*, 4°C, 20 min), the samples were stored at –80°C until further analysis.

Highly sensitive enzyme immunoassays from Salimetrics (State College, USA) were used for cortisol analyses. The procedures were performed according to the manufacturer's specifications. The test has a low sensitivity limit of 0.19 nmol/L. In the present study, the sensitivity ranged from 0.33 to 82.77 nmol/L, and the average intra-assay and inter-assay coefficients of variation were 3.3 and 4.3%, respectively.

### Blood analysis

The day after cortisol evaluation, blood was collected between 6:00 and 8:00 am for BDNF analysis in all subjects and for cotinine analysis in smokers. The blood was collected aseptically by puncturing the median cubital vein, after 8–12 h of fasting and abstaining from cigarettes. The BDNF plasma levels were measured with an ELISA kit (DuoSet®, R&D Systems, USA), according to the manufacturer's instructions. The limit of detection was 5 pg/mL.

To assess the exposure to cigarette smoke and the amount of nicotine absorbed, levels of serum cotinine were measured in smokers. This analysis was performed in a privately-owned laboratory using a chemiluminescence method. A level of cotinine higher than 25 ng/mL was considered the reference value for smokers.

### Statistical analysis

Statistical analyses were performed using the GraphPad Prism 5 (GraphPad Software Inc.®, USA) statistical package. The normality of data was checked by the Shapiro-Wilk test and the homogeneity by the Levene test. As the data were normally distributed, the comparison of results of the light and heavy smokers and the control group was performed by ANOVA and the Scheffé *post hoc* test. Comparison of the smoking history variables between light and heavy smokers was performed with the independent *t*-test. The level of statistical significance was P≤0.05, and data are reported as mean±SD.

## Results

Twenty-seven subjects (13 non-smokers and 14 smokers) completed the study. Four subjects from each group were excluded for not having a normal circadian rhythm. All the light smokers consumed 10 cigarettes/day, and all heavy smokers consumed 20 cigarettes/day. The general characteristics and smoking history of the participants are shown in Table 1. There were no significant differences between groups in terms of age, BMI, body fat percentage, FEV_1_, FVC, FEV_1_/FVC, pack-years and smoking time (P>0.05). In relation to the level of cigarette exposure, heavy smokers presented statistically higher cotinine levels than light smokers (P=0.002).

**Table 1.** General characteristics of smokers and control subjects.

General characteristics	Control (n=13)	Light smoker (n=7)	Heavy smoker (n=7)	P
Age (years)	31.83±4.97	37.43±7.55	34.43±5.35	0.152
BMI (kg/m^2^)	22.22±1.83	21.26±2.14	21.49±2.23	0.618
Body fat (%)	11.60±7.59	10.82±5.48	10.04±7.09	0.909
FEV1 (% predicted)	100.5±4.77	92.8±11.63	94.81±5.35	0.159
FVC (% predicted)	96.43±6.92	95.02±9.64	96.96±7.32	0.895
FEV1/FVC (%)	88.06±4.78	81.89±4.61	82.74±5.55	0.053
Cotinine (ng/mL)	–	86.29±34.74	230.57±90.35	0.002*
Pack-years (number)	–	10.25±4.91	14.60±1.52	0.100
Smoking time (years)	–	21.0±7.18	15.14±5.69	0.129

Data are reported as mean±SD. BMI: body mass index; FEV_1_: forced expiratory volume in 1 s; FVC: forced expiratory volume.

* P<0.05 (*t*-test).

**Figure 1.**
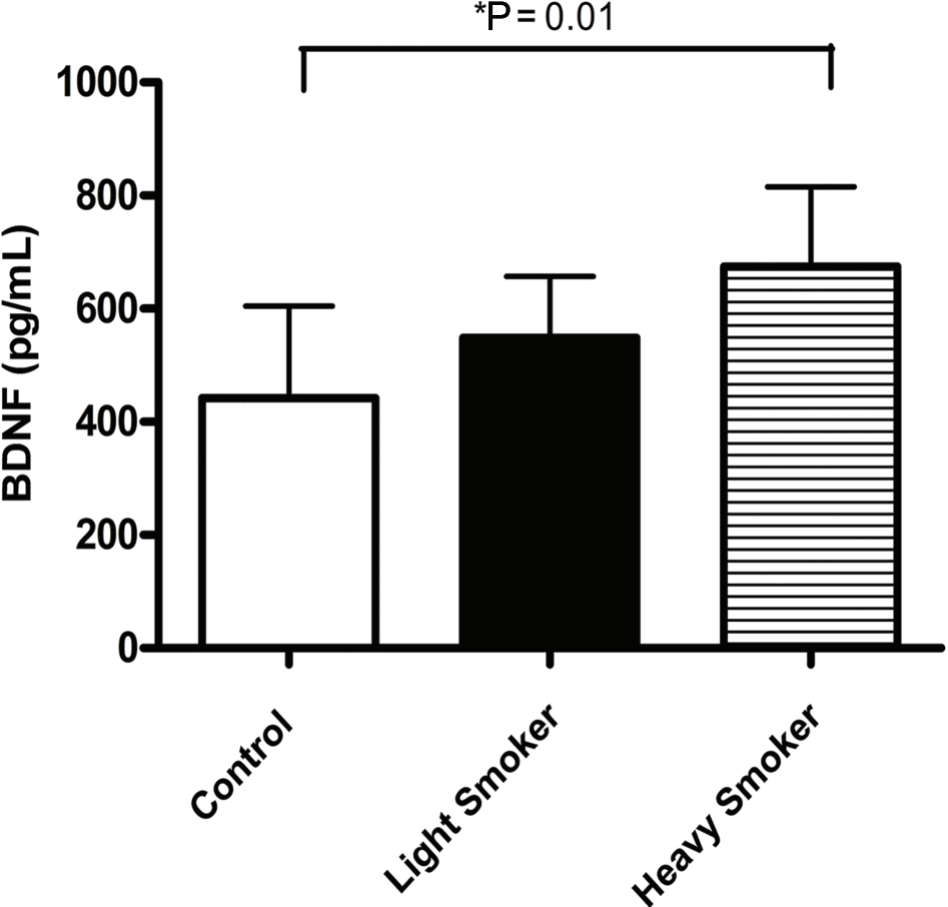
Plasma brain-derived neurotrophic factor (BDNF) levels in control and smoker groups. The plasma BDNF levels in the heavy smokers (674.4±140.7 pg/mL) were significantly higher than that of the control group (441.7±163.0 pg/mL). No difference was observed between light smokers and control and between light and heavy smokers. Data are reported as mean±SD. *P<0.05 (ANOVA).

Plasma BDNF levels are presented in Figure 1. Heavy smokers showed an increase in BDNF levels compared to the control group (P=0.01), whereas this difference was not found in light smokers compared to control. The cortisol release profile during the day is reported in Table 2. Heavy smokers presented significantly lower cortisol levels in the last measurement (10:00 pm) than the control group (P=0.02). Although heavy smokers had lower cortisol levels than the control and light smoker groups, in all the other collections, this difference was not significant.

**Table 2.** Salivary cortisol levels of smokers and control subjects.

Cortisol (nmol/L)	Control (n=13)	Light smoker (n=7)	Heavy smoker (n=7)
Immediately after waking up	15.49±5.59	15.53±5.92	11.05±3.52
30 min after waking up	18.41±7.13	14.73±5.11	14.43±3.97
10:00 am	7.21±1.74	7.66±3.43	6.25±2.10
12:00 am	7.70±3.25	6.71±3.40	6.38±3.30
5:00 pm	3.96±2.11	4.25±2.27	2.80±1.36
10:00 pm	3.45±1.53	2.41±1.34	1.59±0.72*

Data are reported as means±SD.

* P=0.02 compared with control group (ANOVA).

## Discussion

To our knowledge, this is the first study to simultaneously assess BDNF and cortisol levels in healthy smokers. Our data demonstrate that heavy smokers presented an increase in BDNF and a reduction in cortisol levels measured at 10:00 pm compared to non-smokers. These results corroborate other studies in humans that demonstrate a dependence between BDNF and cortisol levels that is related to the amount of smoking (5,8).

Pre-clinical data have shown that nicotine induces BDNF release and upregulates BDNF receptors in the rat brain (4). In humans, there is some evidence that smokers have higher levels of serum BDNF than non-smokers, which is directly related to the amount of cigarette consumption (5). Our data confirm these results, as only heavy smokers showed a significant increase in the quantity of BDNF, suggesting a stimulus-dependence scenario. In this scenario, nicotine increases BDNF and could contribute to mesolimbic synaptic rearrangements, such as in the hypothalamus and hippocampus, involved in the development and maintenance of the smoking habit (4).

Similarly to BDNF, the cortisol response to nicotine seems to be dependent on the frequency of exposure (8). Recently, it was reported that individuals with a lower level of nicotine dependence had a greater cortisol response to a stressor, whereas those with a higher level of nicotine dependence did not show cortisol changes in response to the same stressor (12). It is known that repeated or chronic exposure to nicotine induces the desensitization of the nicotinic acetylcholine receptor (nAChR) in the brain, leading to an increase in the number of receptors with a functional deactivation (13). With respect to our data, the cortisol pattern related to chronic nicotine exposure might have been reduced by desensitization. Although cortisol reduction was significantly different only in the last measurement, this result may have important clinical relevance. The evaluation of salivary cortisol between 10:00 and 11:00 pm, for example, has been one of the main measurements used for the diagnosis of Cushing's syndrome (hypercortisolism).

In addition, the lower cortisol level in heavy smokers could be caused by the stimulation of the mesolimbic system because the activation of the reward circuitry underlying addictive behavior results in the release of excitatory neurotransmitters in brain areas, such as the hippocampus and amygdala (14). Although the hippocampus contains the highest concentration of glucocorticoid receptors, it has been considered to have an inhibitory influence on the HPA axis (15). For example, a lesion of the hippocampus leads to glucocorticoid hypersecretion under basal and stressed conditions (16), and the stimulation of most parts of this structure inhibits stress-induced HPA activation (17). Furthermore, it is believed that repeated activation of the HPA axis in habitual smokers might lead to a down-regulation of corticotropin-releasing hormone (CRH) receptors over time, which could explain the hyporesponsiveness of the HPA axis to a psychosocial or pharmacological challenge (18).

As mentioned above, cortisol modulates dopaminergic activity in the reward system, possibly caused by a negative feedback, because cortisol increases dopamine levels and dopamine receptor stimulation promotes a decrease in cortisol (19). Strengthening this hypothesis, it has been suggested that higher salivary cortisol levels are associated with early smoking relapse in conditions of withdrawal (18), a state that is caused by a decrease in central dopamine levels.

Moreover, although light smokers did not differ from control subjects in the variables assessed, such as BDNF and cortisol, an increase in cigarette consumption could lead to a pattern similar to that of heavy smokers and promote a higher risk for smoking-related diseases. It is believed that plasma nicotine levels are directly influenced by the number of cigarettes smoked per day. This hypothesis was confirmed by the significant difference in cotinine levels in heavy and light smokers. Furthermore, considering that higher BDNF levels were also observed in heavy smokers, it seems that the increase in nicotine exposure could have increased the activation of the mesolimbic system, thereby modifying the dopaminergic activity and decreasing cortisol levels.

This study has limitations, and the results must be interpreted within the context of its design. Although BDNF was evaluated in plasma, studies have demonstrated that peripheral blood can provide an important reservoir of BDNF. In this context, the BDNF content evaluated in serum could be directly influenced by nicotine-lung stimulation because the smooth muscle of the airway is a significant source of BDNF in response to oxidant stress induced by cigarette smoke (20). Moreover, BDNF levels in serum and plasma are highly correlated with BDNF levels in the central nervous system because BDNF is able to cross (influx and efflux) the blood-brain barrier.

Although the present study needs to be confirmed in a large random series of smokers and non-smokers, the consistency of the results observed supports our conclusions. Furthermore, the results of this study provide subsidies for understanding the effect of nicotine consumption on BDNF and cortisol levels in healthy smokers. In summary, levels of BDNF and cortisol measured at 10:00 pm appear to be dependent on cigarette smoking and could represent the relationship between the mesolimbic system and the HPA axis in the mechanism of nicotine addiction. These results bring new perspectives for the investigation of this mechanism and, consequently, of pharmacological interventions.
